# The Evaluation of the Risk Factors for Non-Muscle Invasive Bladder Cancer (NMIBC) Recurrence after Transurethral Resection (TURBt) in Chinese Population

**DOI:** 10.1371/journal.pone.0123617

**Published:** 2015-04-07

**Authors:** Shenghua Liu, Junyao Hou, Hu Zhang, Yishuo Wu, Mengbo Hu, Limin Zhang, Jianfeng Xu, Rong Na, Haowen Jiang, Qiang Ding

**Affiliations:** 1 Fudan Institute of Urology, Huashan Hospital, Fudan University, Shanghai, China; 2 Department of Urology, Huashan Hospital, Fudan University, Shanghai, China; 3 State Key Laboratory of Genetic Engineering, School of Life Science, Fudan University, Shanghai, China; 4 Center for Cancer Genomics, Wake Forest School of Medicine, Winston-Salem, NC, United States of America; Nanjing Medical University, CHINA

## Abstract

**Objective:**

The risk factors of bladder cancer recurrence after transurethral resection of bladder tumor (TURBt) were poorly understood, especially in Chinese population. This study evaluated the potential risk factors of recurrence based on a Chinese population.

**Materials and Methods:**

A total of 698 patients that received TURBt procedure in our institute from 2000 to 2012 were recruited in this study. Clinical information was collected. The patients were followed up according to the schedule recommended by Chinese guideline.

**Results:**

A total of 583 males (83.5%) and 115 females (16.5%) were enrolled in our study. The median follow-up duration was 51.5 months. Gender, chief complain, tumor size, number of lesions, histological grade and chemotherapeutic agents were found significantly associated with patients’ short-term recurrence (less than 1 year) (All p<0.05). In the multivariate analysis, tumor size, number of lesions, histological grade and chemotherapeutic agents were significantly related to patients’ short-term recurrence (less than 1 year) (All p<0.05). A multivariate model based on tumor size, number of lesions, histological grade and chemotherapeutic agents had an AUC of 0.697, which significantly improved the prediction utility for bladder cancer short-term recurrence (less than 1 year) than any single factor In the multivariate Cox regression, tumor size greater than 3 cm, multifocal lesions, worsen histological grade and non-urothelial carcinoma was related to time to recurrence (TR).

**Conclusion:**

Patients with larger tumor size, multifocal number of lesions, higher tumor grade and who received chemotherapeutic agents other than Epirubicin and Pirarubicin might have higher risks of recurrence less than 1 year. Tumor size, number of lesions, pathology and histological grade might be associated with TR. As Bacille Calmette-Guerin (BCG) is currently not approved for bladder cancer in China, Epirubicin and Pirarubicin might be considered prior to other chemotherapy medications when providing post-operative instillation of chemotherapy.

## Introduction

Urinary bladder cancer remains one of the most commonly diagnosed cancers in the world. It was estimated that 74,690 new cases and 56,390 deaths from bladder cancer occurred in 2014 in U.S.[1]. In China, the incidence rate of bladder cancer was the 8^th^ highest among all the cancers in 2007[2] Approximately 75% of newly diagnosed bladder cancers were non-muscle invasive, including tumor confined to mucosa (Ta), submucosa (T1), and carcinoma in situ (CIS)[3]. Transurethral resection (TUR) followed by intravesical instillation is the standard therapy for non-muscle invasive bladder tumor (NMIBC).

Despite the treatment effort, NMIBC is considered as a “troublesome” disease with relatively high possibility to recur. The recurrence rate for NMIBC in 1 year was reported ranging from 15% to 70%[4], of which 7% to 40% were found to progress within 5 years[5]. Therefore, a regular follow-up after the surgery should be as equally important as the resection of primary tumor. The guidelines suggested a follow-up schedule of undertaking cystoscopy every 3 months for the first two year, and every 6 months thereafter[6]. Considering that a proportion of the NMIBC patients actually did not recur within the first year after the operation[4], such frequency of undertaking cystoscopy might be a burden for patients as well as health insurance system worldwide. Therefore, researchers were trying to evaluate every potential risk factor for predicting the recurrence of NMIBC. For instance, external exposure like smoking, high body mass index[7]; Tumor characteristics like tumor size, invasion depth, tumor grade, presence of carcinoma in situ[8,9,10]; Adjuvant treatment like a single immediate post-operative instillation of chemotherapy and the presence of adjuvant instillation[8,11], are the risk factors that account for the recurrence of NMIBC. Several studies have validated the practice of different scoring models based on these risk factors [12,13,14]. The most popular model are the EORTC model published by European Organization for Research and Treatment Of Cancer (EORTC) Genito-Urinary Group in 2006[15] and CUETO model published by the Club Urolo ´gico Espan ´ol de Tratamiento Oncolo ´gico (CUETO) in 2009[16].

However, only a few data were reported evaluating EORTC and CUETO in Chinese population[13]and the performance of the prediction tools was not as good as it performed in Caucasian population. In addition, Bacille Calmette-Guerin (BCG) which is commonly used as adjuvant intravesical therapy medication after TUR in western countries has not been approved for bladder cancer by China Food and Drug Administration (CFDA). Therefore, predicting tools based on Caucasian population might not be suitable for Chinese population. In this study, our object was to evaluate the potential risk factors of NMIBC recurrence after TUR in Chinese population.

## Materials and Methods

### Study population

Medical records of all the patients (n = 990) who were diagnosed as bladder cancer from January 2000 to December 2012 in Huashan Hospital, Fudan University, Shanghai, China were reviewed. Huashan Hospital is a tertiary hospital in Shanghai, China. Patients from all over the country seek their service because of its high quality of medical health care. The inclusion criteria were: 1) TUR was performed as initial treatment; 2) Tumor was diagnosed as NMIBC; 3) The patient who received adjuvant intravesical chemotherapy after TUR. The chemotherapy drugs included the anthracycline antibiotic like Epirubicin (EPI) or Pirarubicin (THP), Mitoxantrone (MTZ), Mitomycin C (MMC) and hydroxycamptothecine. None of the patients had adjuvant immunotherapy due to the inaccessible of BCG in China. The exclusion criteria were 1) patients with missing pathological information; 2) patients failed to receive further treatment because of severe complications. For each patient, the following clinical factors were collected: age, gender, number of tumors, tumor size, pathological classification, tumor grade, immediate post-operative instillation of chemotherapy and chemotherapeutic agents. Tumor grade was re-evaluated according to the 1973 WHO classification [17] by single group of pathology doctors in Pathology Department in our institute.

Patients were required to be followed-up by rigid cystoscopy every 3 month for 2 years, the every 6 month until 5 years and annually thereafter according to the Chinese guideline exactly the same as US and European guidelines[6]. All the patients in the study at least completed the first two year follow-up regime. Recurrence was defined as a histologically confirmed newly-emerging tumor after complete resection of NMIBC. Short-term recurrence was defined as recurrence happened within the first year of follow-up. The study was approved by institutional review board of Huashan Hospital, Fudan Unviersity, Shanghai, China. Written informed consent was obtained from each patient. All data was anonymized before being used in this study.

### Statistical analysis

A descriptive study of all the variables was conducted. All patients were categorized into two groups: 1) short-term recurrence (recurrence happened within 1 year after TURBt) 2) non-short-term recurrence (no recurrence and time to recurrence longer than 1 year after TURBt during follow-up). For statistical analysis, we used the Chi-square test to compare categorical variables between short-term and non-short-term recurrence group, the paired t-test for continuous parametric variables. The univariate logistic regression was used to evaluate differences among groups. Selected variables that showed significant differences were included in a multivariate logistic regression analysis to further identify predictive parameters of short-term recurrence. The predicted probability of short-term recurrence from multivariate logistic model was used as a surrogate marker to construct receiver operating characteristic (ROC) curve. Area under curve (AUC) of ROC was used to evaluate the predictive performance of short-term recurrence. Z-test was performed to evaluate the difference among AUCs of each factors and the model. To determine effect of the different variables on time to recurrence (TR, as a continuous vairable), univariate and multivariate analysis were performed using Cox proportional hazards model with stepwise forward selection. Differences were considered significant if p<0.05. All statistical analysis was performed using IBM SPSS version 19.0 (SPSS, IBM company, Armonk, New York).

## Results

A total of 698 patients were enrolled in our study with 583 males (83.5%) and 115 females (16.5%). The characteristics of the patients were shown in Table 1. The mean age at diagnosis was 63.7±14.0. The median follow up duration was 51.5 months (ranged from 24 to 245 months). Of all the patients, 106 (15.2%) suffered recurrence within 1 year. Older (older age at diagnosis) and female patients were more likely to recur within 1 year (p = 0.035 and 0.007 respectively, Table 1). In addition, chief complain, tumor size, number of lesions, locations, tumor grade and chemotherapeutic agents were significantly associated with early recurrence (all p<0.05). 205 out of 698 patients (29.4%) received immediate post-operation intravesical chemotherapy within 24 hours after TUR. However, no significant association was observed between immediate instillation and early recurrence (p = 0.354). No significant difference of pathological pattern was observed between short-term recurrence group and non-short-term recurrence group (p = 0.424).

**Table 1:** Clinical characteristics of all the patients and comparison of each variable between short-term and non-short-term recurrence group.

	Overall	Non-short-term recurrence(>1year)	Short-term recurrence (≤1 year)	p
Patients (n, %)	698	592(84.8)	106(15.2)	
Age(year)				**0.035**
Mean±SD	63.7±14.0	63.2±14.0	66.2±13.8	
Gender (n, %)				**0.007**
Male	583(83.5)	504(84.8)	79(74.5)	
Chief Complain (n, %)				**0.002**
With symptoms	588(84.2)	498(84.1)	90(84.9)	
Without symptoms	82(11.7)	77(13.0)	5(4.7)	
Missing	28(4.1)	17(2.9)	11(10.4)	
Size(n, %)* ^1^				**0.004**
<3cm	411(58.9)	368(62.2)	43(40.6)	
≥3cm	244(35.0)	199(33.6)	45(42.5)	
Missing	43(6.1)	25(4.2)	18(17.0)	
Number of Lesions^1^ (n, %)				**0.001**
Single	417(59.7)	377(63.7)	40(37.7)	
2–7	177(25.4)	146(24.6)	31(29.2)	
≥8	70(10.0)	53(9.0)	17(16.0)	
Missing	34(4.9)	16(2.7)	18(17.0)	
Location (n, %)				**0.024**
With trigone	76(10.9)	63(10.6)	13(12.3)	
With neck	65(9.3)	54(9.1)	11(10.4)	
With trigone&neck	18(2.6)	12(2.0)	6(5.7)	
Without trigone or neck	506(72.5)	448(75.7)	58(54.7)	
Missing	33(4.7)	15(2.5)	18(17.0)	
Pathology (n, %)				0.424
Urothelial Carcinoma	685(98.1)	582(98.3)	103(97.2)	
Others	13(1.9)	10(1.7)	3(2.8)	
Grade (n, %)				**0.001**
Urothelial papilloma	11(1.6)	11(1.9)	0(0.0)	
1	120(17.2)	110(18.6)	10(9.4)	
2	426(61.0)	367(62.0)	59(55.7)	
3	113(16.2)	82(13.9)	31(29.2)	
Missing	28(4.0)	22(3.7)	6(5.7)	
Immediate instillation (n, %)				0.354
<24h	205(29.4)	182(30.7)	23(21.7)	
≥24h	418(59.9)	360(60.8)	58(54.7)	
Misssing	75(10.7)	50(8.4)	15(14.2)	
Chemotherapeutic agents** (n, %)				**0.001**
EPI or THP	482(69.1)	423(71.5)	59(55.7)	
Others	216(30.9)	169(28.5)	47(44.3)	

* Size: the diameter of the largest lesion;

** Chemotherapeutic agents: the chemotherapy medications that were used in post-operative instillation

^1^ The categorizations was the same as the 2013 EAU guideline

We performed further analysis by using logistic regression to evaluate each variable (S1 Table). Gender (OR = 1.957, p = 0.007), chief complain (OR = 0.359, p = 0.031), tumor size (OR = 1.935, p = 0.004), number of lesions (OR = 1.784, p<0.001), histological grade (OR = 2.189, p<0.001) and chemotherapeutic agents (OR = 1.994, p = 0.001) were significant factors associated with short-term recurrence.

The above significant factors were put into multivariate logistic regression analysis to further evaluate significant clinical variables with early recurrence (Table 2). Factors significant in the univariate analysis were adjusted in the multivariate model. Early recurrence was significantly associated with tumor size (≥3cm vs. others, OR = 1.751, p = 0.026) and number of lesions (lesions≥8 vs. others, OR = 3.035, p = 0.001). Histological grades 3 tumors were more likely to recur in 1 year (OR = 2.870, p = 0.015). Comparing with other chemotherapeutic agents, patients using EPI or THP were less likely to recur in 1 year (OR = 1.835, p = 0.021). A prediction model was then built based on the results of the multivariate logistic regression to predict the risk of short-term recurrence including the risk factors of tumor size, number of lesions, histological grades and chemotherapeutic agents.

**Table 2:** Multivariate logistic regression analysis evaluating the risk factors for short-term recurrence.

variable		OR	95% CI	p-value
Gender		1.774	0.982–3.204	0.057
Chief complain		0.577	0.219–1.521	0.267
Size		1.751	1.070–2.866	**0.026**
Number of lesions	single	1.000(Ref)	Ref	Ref
	2–7	1.715	0.989–2.974	0.055
	> = 8	3.035	1.537–5.995	**0.001**
Grade	0	0	0	0
	1	1.000(Ref)	Ref	Ref
	2	1.326	0.615–2.859	0.472
	3	2.870	1.223–6.730	**0.015**
Chemotherapeutic agents		1.835	1.094–3.080	**0.021**

OR: odds ratio; CI: confidence interval

**Fig 1:**
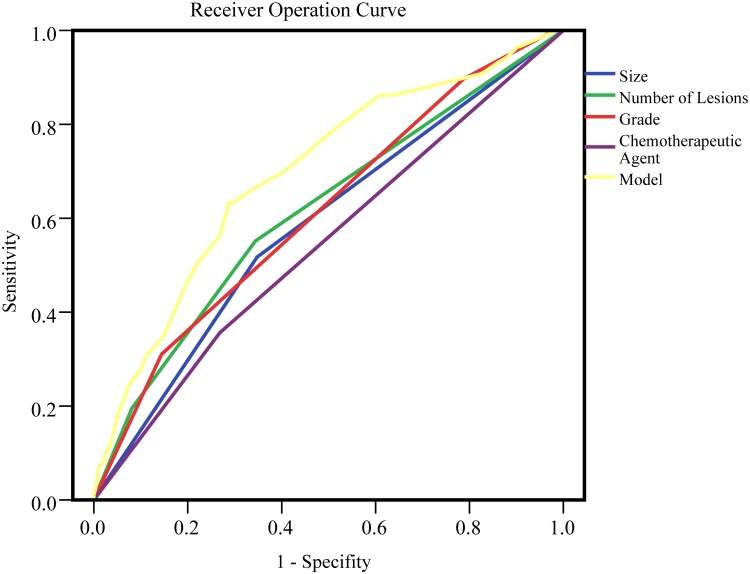
Receiver operating curve of short-term recurrence. Receiver operating curve of short-term recurrence includes clinical variables as size (AUC = 0.585), number of lesions(AUC = 0.615), grade(AUC = 0.613), chemotherapeutic agents(AUC = 0.544) and the multi-variate model(AUC = 0.697).

To evaluate the performance of each risk factor and the model for predicting short-term recurrence of NMIBC, ROC analysis was performed. (Table 3, Fig 1). The AUCs of tumor size, number of lesions, histological grade and chemotherapeutic agents were 0.585, 0.615, 0.613 and 0.544 respectively. The AUC of multivariate prediction model was 0.697, which was significantly outperformed any single risk factor.

**Table 3:** Area under receiver operating curve.

variable	Area under curve	P-value^†^
Size	0.585	**0.019**
Number of lesions	0.615	0.082
Grade	0.613	0.071
Chemotherapeutic agents	0.544	**0.002**
Multi-variate Model*	0.697	

*The multi-variate model was built based on the variables of size, number of lesions, grade, chemotherapeutic agents.

^†^P-values presented the differences between Multi-variate Model and other variables.

Cox regression was performed to evaluate the association between each risk factor and TR as a continuous variable (not categorized into short-term and non-short-term groups). In the current study, 203 (29.1%) patients experienced recurrence within the observation period. In univariate Cox regression (Table 4), with symptoms at onset, tumor size greater than 3 cm, multifocal lesions, worsen pathological grade and using EPI or THP as chemotherapeutic agents were significantly associated with TR (all p<0.05). Multivariate Cox regression was performed adjusting for the following variables: age, gender, chief complain, tumor size, location, number of lesions, pathological classification, histological grade, immediate post-operative instillation of chemotherapy and chemotherapeutic agents (Table 4). Tumor size greater than 3 cm (RR (relative risk) = 1.496, p = 0.014), multifocal lesions (RR = 3.414, p<0.001), worsen histological grade (RR = 2.473, p = 0.002) and non-urothelial carcinoma (RR = 11.270, p<0.001) were found to be associated with TR significantly.

**Table 4:** Univariate and multivariate cox regression analysis for recurrence.

Factors*	Univariate		Multivariate	
	RR (95% CI range)	p-value	RR (95% CI range)	p-value
Age	1.009(0.999–1.020)	0.088		
Gender	1.088(0.755–1.567)	0.652		
Chief complain	0.412(0.224–0.759)	**0.004**		
Size	1.681(1.234–2.289)	**0.001**	1.496(1.085–2.063)	**0.014**
Number of Lesions				
Single	1.000(Ref)	Ref	1.000(Ref)	Ref
2–7	2.069(1.476–2.900)	**<0.001**	1.867(1.293–2.694)	**0.001**
>=8	3.348(2.138–5.243)	**<0.001**	3.414(2.103–5.541)	**<0.001**
Location	0.957(0.834–1.098)	0.533		
Pathology	1.788(0.734–4.352)	0.201	11.270(3.391–37.459)	**<0.001**
Grade				
Urothelial papilloma	0	0	0	0
1	1.000(Ref)	Ref	1.000(Ref)	Ref
2	1.770(1.414–2.744)	**0.011**	1.524(0.933–2.487)	0.092
3	3.247(1.965–5.368)	**<0.001**	2.473(1.400–4.367)	**0.002**
Immediate instillation	1.206(0.843–1.726)	0.305		
Chemotherapeutic agents	1.370(1.029–1.824)	**0.031**		

*203 out of 698 patients experience recurrence within the follow-up periods.

RR: relative risk; CI: confidence interval

## Discussion

Bladder tumor has a high tendency to recur despite TUR and adjuvant intravesical chemotherapy. Thus, it is of clinical importance to identify the patients with relatively higher recurrent potential in early stage so that more frequent follow-up or early radical cystectomy might be recommended. Therefore, in this study, we aimed to figure out the risk factors which could predict the potential recurrence, especially short-term recurrence (less than 1 year) after TURBt.

In our study, tumor size, multifocal lesions and histological grade were found significantly associated with short-term recurrence and TR. The results were similar to other studies[5,18]. Larger size and more lesions imply greater tumor burden and harder complete removal of the tumor via TURBt. Another important risk factor of tumor recurrence is the biological characteristics. Multi-focality and recurrence of transitional cell carcinoma showed a general genetic instability in the entire transitional epithelium[19]. We did not evaluate the biological risk factors because of the inconveniences of the testing. In the current study, we would like to provide an easily used prediction tool.

Another finding in our study was that chemotherapeutic agents were significantly related to patients’ short-term recurrence. Although BCG instillation after TUR remains the best choice [6]. chemotherapy instillation is the only option in China currently. Our study revealed that anthracycline antibiotics (e.g. EPI and THP) outweighed other agents,(e.g. Mitoxantrone (MTZ), Mitomycin C (MMC) and hydroxycamptothecine) in preventing short-term recurrence. The effect of EPI has been proved in previous study [20]. Given the limited prospective studies comparing two different chemotherapeutic agents, there is still no conclusion that which chemotherapeutic agents would lead to a better outcome. Our study indicated that EPI or THP should be firstly considered for instillation chemotherapy after TURBt in China. Although the conclusion should be validated in prospective studies, it might provide evidence for clinical practice in China.

When evaluated TR, some of the factors such as chemotherapeutic agents failed to show its influence, which was significantly associated with short term recurrence. As showed in Table 4, only tumor size, number of lesions, pathology and tumor grade were significantly associated with TR. Those factors were related to characteristics of tumors. It might indicate that although exogenous efforts like intravesicle instillation could delay the time to first recurrence, tumor inherent biological characteristics played the crucial roles in long term prognosis.

In the current study, we hoped to establish a prediction tool for short-term recurrence of NMIBC after TURBt. The common prediction tools are nomograms and risk calculators. Since nomograms are usually difficult to apply in daily practice, we chose to establish a risk prediction model based on the different weights of the variables from the multivariate logistic regression result. In the current study, tumor size, multifocal lesions, histological grade and chemotherapeutic agents were found significant in the multivariate analysis after adjusting for other variables. So our model was built based on the above variables. This model would have added value for predicting early recurrence of NMIBC after TURBt with an AUC of 0.697. The merit of the prediction model was that it was all based on clinical factors, so it would be easy for clinical practitioner to use if a risk calculator could be further evaluated in validation studies.

Whether early intravesical chemotherapy within 24 hours after TURBt could decrease the risk of recurrence is still under debate[11,21,22]. We did not observe the association between early intravesical instillation of chemotherapy and recurrence in the current study. As for the limitation of sample size, further randomized prospective studies should be performed to evaluate whether an early intravesical instillation of chemotherapy medication is benefit for post-TURBt NMIBC in Chinese population.

There remained to be several limitations in our study. First, the study was retrospective from one institution, which might conduct selection bias. However, as mentioned before, patients in our institute may represent Chinese population, especially population from southeast part of China. Second, our follow-up was not long enough to meet the outcome of mortality. Although we were not able to evaluate the association between risk factors and survival, the main purpose of this study was to access the risk factors of cancer recurrence. The results from the study might be used for individualizing the follow-up plan of post-TURBt patient. Finally, there might be limitation of our model since it was built based on the population of current study, the result might be over-expected. Thus, the prediction performance of the model still needs to be validated in another independent population.

## Conclusion

To conclude, patients with larger tumor size, multifocal number of lesions, higher tumor grade might be considered having highest risk of recurrence within 1 year in Chinese population. These risk factors should be comprehensively evaluated when predicting the probability of short-term recurrence of NMIMC. Further studies based on larger populations may be performed to help individualize the follow-up plan for each patient based on these risk factors. In addition, because of the inaccessibility of BCG in China, anthracycline antibiotics (EPI and THP) should be prior considered for intravesical chemotherapy.

## Supporting Information

S1 Table Univariate logistic regression analysis for evaluating the risk factors for short-term recurrence. Gender (OR = 1.957, p = 0.007), chief complain (OR = 0.359, p = 0.031), tumor size (OR = 1.935, p = 0.004), number of lesions (OR = 1.784, p<0.001), histological grade (OR = 2.189, p<0.001) and chemotherapeutic agents (OR = 1.994, p = 0.001) were significant factors associated with short-term recurrence.
(DOC)
