# Supplementary material for: The Evaluation of the Risk Factors for Non-Muscle Invasive Bladder Cancer (NMIBC) Recurrence after Transurethral Resection (TURBt) in Chinese Population
Source: PLoS One. 2015 Apr 7;10(4):e0123617. doi: 10.1371/journal.pone.0123617 (PMC4388336; doi:10.1371/journal.pone.0123617)
Supplement: S1 Table — Gender (OR = 1.957, p = 0.007), chief complain (OR = 0.359, p = 0.031), tumor size (OR = 1.935, p = 0.004), number of lesions (OR = 1.784, p<0.001), histological grade (OR = 2.189, p<0.001) and chemotherapeutic agents (OR = 1.994, p = 0.001) were significant factors associated with short-term recurrence. (DOC) [file pone.0123617.s001.doc]

**Table S1. Univariate logistic regression analysis for evaluating the risk factors for short-term recurrence**

| variable | OR | 95% CI | p-value |
| --- | --- | --- | --- |
| age | 1.015 | 0.9999-1.031 | 0.071 |
| Gender | 1.957 | 1.196-3.201 | **0.007** |
| Chief complain | 0.359 | 0.141-0.912 | **0.031** |
| size | 1.935 | 1.231-3.041 | **0.004** |
| Number of lesions | 1.784 | 1.323-2.406 | **<0.001** |
| Location | 0.834 | 0.687-1.011 | 0.065 |
| pathology | 1.695 | 0.459-6.264 | 0.426 |
| Grade | 2.189 | 1.524-3.146 | **<0.001** |
| Immediate instillation | 1.264 | 0.756-2.116 | 0.372 |
| Chemotherapeutic agents | 1.994 | 1.307-3.043 | **0.001** |

OR: odds ratio; CI: confidence interval
